# Supplementary material for: Deletion of the Autism-Associated Protein SHANK3 Abolishes Structural Synaptic Plasticity after Brain Trauma
Source: Int J Mol Sci. 2022 May 29;23(11):6081. doi: 10.3390/ijms23116081 (PMC9181590; doi:10.3390/ijms23116081)
Supplement: Supplementary file 1 [file ijms-23-06081-s001.zip › ijms-1699987-Supplementary.pdf]

International Journal of *Molecular Sciences*

MDPI

Supplementary Information

Deletion of the Autism-Associated Protein Shank3 Abolishes Synaptic Plasticity After Brain Trauma

Carolina Urrutia-Ruiz, Daniel Rombach, Silvia Cursano, Susanne Gerlach-Arbeiter, Michael Schoen, Juergen Bockmann, Maria Demestre and Tobias M Boeckers

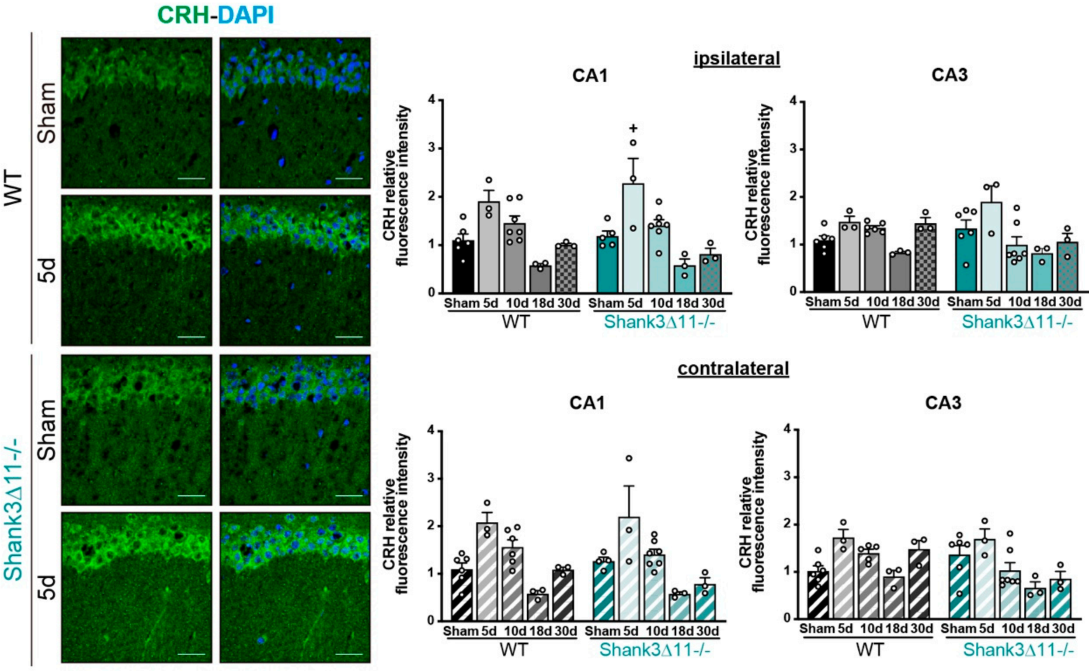

Figure S1. Corticotropin-Releasing Hormone (CRH) expression slightly increased in *Shank3Δ11-/-* animals following mTBI. CRH immunostaining (green) and DAPI (blue) for the ipsilateral and contralateral CA1 and CA3 hippocampus. Representative images of the ipsilateral CA1 region (scale bar 20 m) (left panel) of WT sham and after five dpi (upper left panel) and *Shank3Δ11-/-* sham and after five dpi. CRH fluorescence quantification in the CA1 and CA3 hippocampus on the ipsilateral hippocampus in WT (filled black bars) and *Shank3Δ11-/-* mice (filled turquoise bars) (right panel), and for the contralateral side in WT (hatched black bars) and *Shank3Δ11-/-* mice (hatched turquoise bars). N=3-7. Data are shown as mean ± SEM. Data sets were analyzed using two-way ANOVA (genotype x mTBI) and Bonferroni's post hoc comparison test. [Ipsilateral CA1: genotype $F(1, 33) = 0.1422$, $p = 0.7085$; mTBI $F(4, 33) = 15.73$, $p < 0.0001$; genotype x mTBI $F(4, 33) = 0.5429$, $p = 0.7053$. Ipsilateral CA3: genotype $F(1, 34) = 0.01073$, $p = 0.9181$; mTBI $F(4, 34) = 5.528$, $p = 0.0015$; genotype x mTBI $F(4, 34) = 1.973$, $p = 0.1209$]; [Contralateral CA1: genotype $F(1, 31) = 0.06393$, $p = 0.8021$; mTBI $F(4, 31) = 14.55$, $p < 0.0001$; genotype x mTBI $F(4, 31) = 0.4763$, $p = 0.7528$. Contralateral CA3: genotype $F(1, 33) = 2.081$, $p = 0.1586$; mTBI $F(4, 33) = 1.774$, $p = 0.1576$; genotype x mTBI $F(4, 33) = 1.774$, $p = 0.1576$] (+ $p < 0.05$: comparison regarding *Shank3Δ11-/-* sham animals).

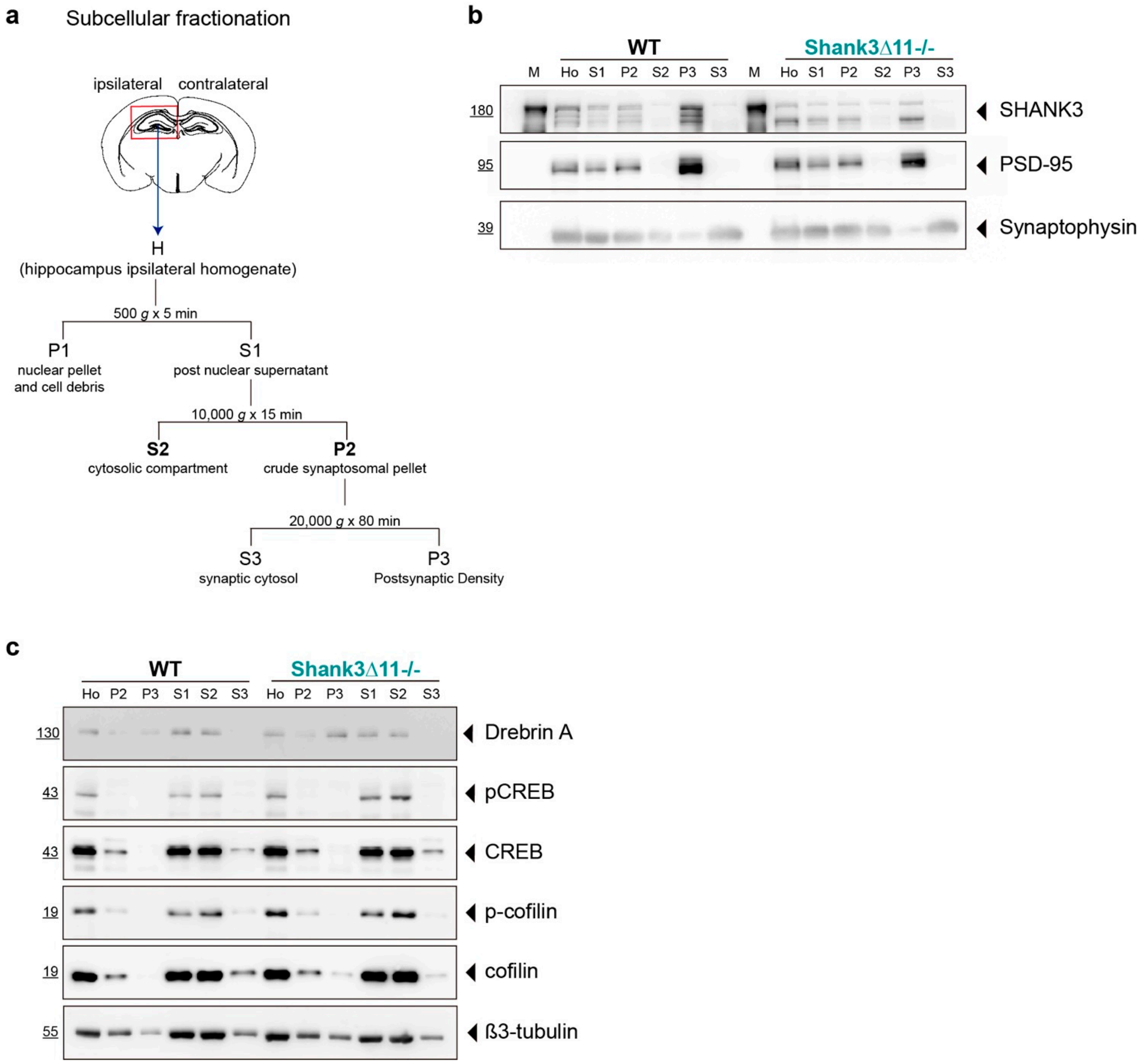

Figure S2. Scheme of differential subcellular fractionation steps and a screening performed for synaptic plasticity-related proteins. (a) Diagram of the differential centrifugation conducted in the ipsilateral hippocampus. In bold are the fractions used in this study. (b) Representative western blots of sham WT and *Shank3Δ11*-/- animals assessing the quality of the subcellular fractionation. M (protein ladder). (c) Western blot was performed to screen the enrichment of different structural and functional synaptic plasticity proteins in the fractions obtained from the differential centrifugation.

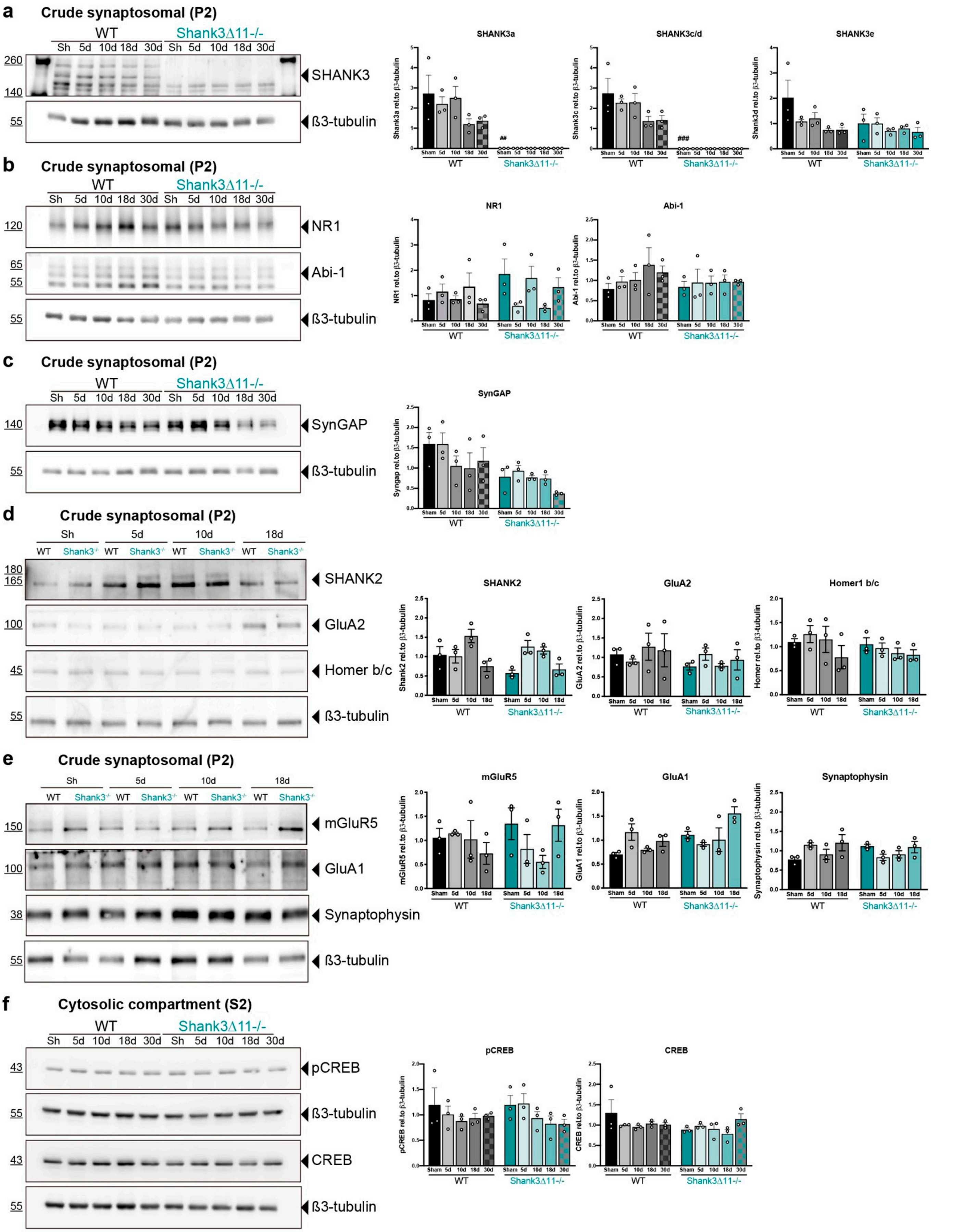
a
Crude synaptosomal (P2)
WT
Shank3Δ11-/-
Sh 5d 10d 18d 30d Sh 5d 10d 18d 30d
260
140
SHANK3
55
ß3-tubulin
SHANK3a
Shank3a rel.to β3-tubulin
##
SHANK3c/d
Shank3c rel.to β3-tubulin
###
SHANK3e
Shank3d rel.to β3-tubulin
Sham 5d 10d 18d 30d
b
Crude synaptosomal (P2)
120
NR1
65
55
Abi-1
NR1 rel.to β3-tubulin
Abi-1 rel.to β3-tubulin
c
Crude synaptosomal (P2)
140
SynGAP
Syngap rel.to β3-tubulin
d
Crude synaptosomal (P2)
Sh 5d 10d 18d
WT Shank3-/-
180
165
SHANK2
100
GluA2
45
Homer b/c
Shank2 rel.to β3-tubulin
GluA2 rel.to β3-tubulin
Homer1 b/c
Homer rel.to β3-tubulin
e
Crude synaptosomal (P2)
150
mGluR5
GluA1
38
Synaptophysin
mGluR5 rel.to β3-tubulin
GluA1 rel.to β3-tubulin
Synaptophysin rel.to β3-tubulin
f
Cytosolic compartment (S2)
43
pCREB
CREB
pCREB rel.to β3-tubulin
CREB rel.to β3-tubulin

Figure S3. Synaptic plasticity related-proteins presented similar expression following mTBI in both wild-type and *Shank3Δ11-/-* mice. (**a**) Western blot analysis of the crude synaptosomal pellet (P2) of the ipsilateral hippocampus for SHANK3 isoforms (left panel) and the quantification of its expression relative to 3-tubulin (right panel) for WT (black bars) and *Shank3Δ11-/-* mice (turquoise bars). N=3. All data are shown as mean ± SEM. All Data sets were analyzed using two-way ANOVA (genotype x mTBI) and Bonferroni's post hoc comparison test. [SHANK3a: genotype F(1, 20) = 72.44, p < 0.0001, mTBI F(4, 20) = 1.656, p = 0.1995; genotype x mTBI F(4, 20) = 1.656, p = 0.1995. SHANK3c/d: genotype F(1, 20) = 113.5, p < 0.0001; mTBI F(4, 20) = 2.009, p = 0.1319; genotype x mTBI F(4, 20) = 2.009, p = 0.1319. SHANK3f: genotype F(1, 20) = 3.304, p = 0.0841; mTBI F(4, 20)= 2.529, p = 0.0727; genotype x mTBI F(4, 20) = 1.223, p = 0.3324] (**b**) Western blot analysis of the crude synaptosomal pellet (P2 fraction) of the ipsilateral hippocampus for NR1 and Abi-1 (left panel) and the quantification of its expression relative to 3-tubulin (right panel) for WT (black bars) and *Shank3Δ11-/-* mice (turquoise bars). N=3. [NR1: genotype F(1, 20) = 0.9781, p = 0.3345; mTBI F(4, 20) = 0.7158, p = 0.5910; genotype x mTBI F(4, 20) = 3.055, p = 0.0407]; [Abi-1: genotype F(1, 20) = 1.032, p = 0.3218; mTBI F(4, 20) = 0.8046, p = 0.5366; genotype x mTBI F(4, 20) = 0.3824, p = 0.8186], (**c**) Western blot analysis of the crude synaptosomal pellet (P2 fraction) of the ipsilateral hippocampus for SynGAP and the quantification of its expression relative to 3-tubulin (left panel) [SynGAP: genotype F(1, 20) = 14.67, p = 0.0010; mTBI F(4, 20) = 1.673, p = 0.1957; genotype x mTBI F(4, 20) = 0.7040, p = 0.5985]. N=3. (**d**) Western blot analysis of the crude synaptosomal pellet (P2 fraction) of the ipsilateral hippocampus for SHANK2, GluA2 and Homer1 b/c and the quantification of its expression relative to 3-tubulin (right panel) for WT (black bars) and *Shank3Δ11-/-* mice (turquoise bars). N=3. [SHANK2: genotype F(1, 16) = 2.531, p = 0.1312; mTBI F(3, 16) = 7.428, p = 0.0025; genotype x mTBI F(3, 16) = 2.286, p = 0.1177], [GluA2: genotype F(1, 16) = 1.715, p = 0.2088; mTBI F(3, 16) = 0.1319, p = 0.9402; genotype x mTBI F(3, 16) = 0.7744, p = 0.5252] , and [Homer1 b/c: genotype F(1, 16) = 1.492, p = 0.2396; mTBI F(3, 16) = 1.357, p = 0.2916; genotype x mTBI F(3, 16) = 0.5472, p = 0.6571]. (**e**) Western blot analysis of the crude synaptosomal pellet (P2 fraction) of the ipsilateral hippocampus for mGluR5, GluA1, and synaptophysin (left panel) and the quantification of its expression relative to 3-tubulin (right panel) for WT (black bars) and *Shank3Δ11-/-* mice (turquoise bars). N=3. [mGluR5: genotype F(1, 16) = 0.01220, p = 0.9134; mTBI F(3, 16) = 0.8079, p = 0.5078; genotype x mTBI F(3, 16) = 1.761, p = 0.1950], [GluA1: genotype F(1, 16) = 6.784, p = 0.0192; mTBI F(3, 16) = 3.643, p = 0.0356; genotype x mTBI F(3, 16) = 3.990, p = 0.0268],[Synaptophysin: genotype F(1, 16) = 0.07776, p = 0.7839; mTBI F(3, 16) = 1.706, p = 0.2060; genotype x mTBI F(3, 16) = 2.933, p = 0.0653]. (**f**) Western blot analysis of the cytosolic compartment (S2 fraction) of the ipsilateral hippocampus for pCREB and CREB (left panel) and the quantification of its expression relative to 3-tubulin (right panel) for WT (black bars) and *Shank3Δ11-/-* mice (turquoise bars). N=3. [pCREB: genotype F(1, 20) = 0.000, p > 0.9999; mTBI F(4, 20) = 1.532, p = 0.2310; genotype x mTBI F(4, 20) = 0.3939, p = 0.8106], [CREB :genotype F(1, 20) = 2.182, p = 0.1552; mTBI F(4, 20) = 10.8836, p = 0.4915; genotype x mTBI F(4, 20) = 1.458, p = 0.2520]. (## p < 0.01; ### p < 0.001: comparison between WT sham and *Shank3Δ11-/-* sham animals).

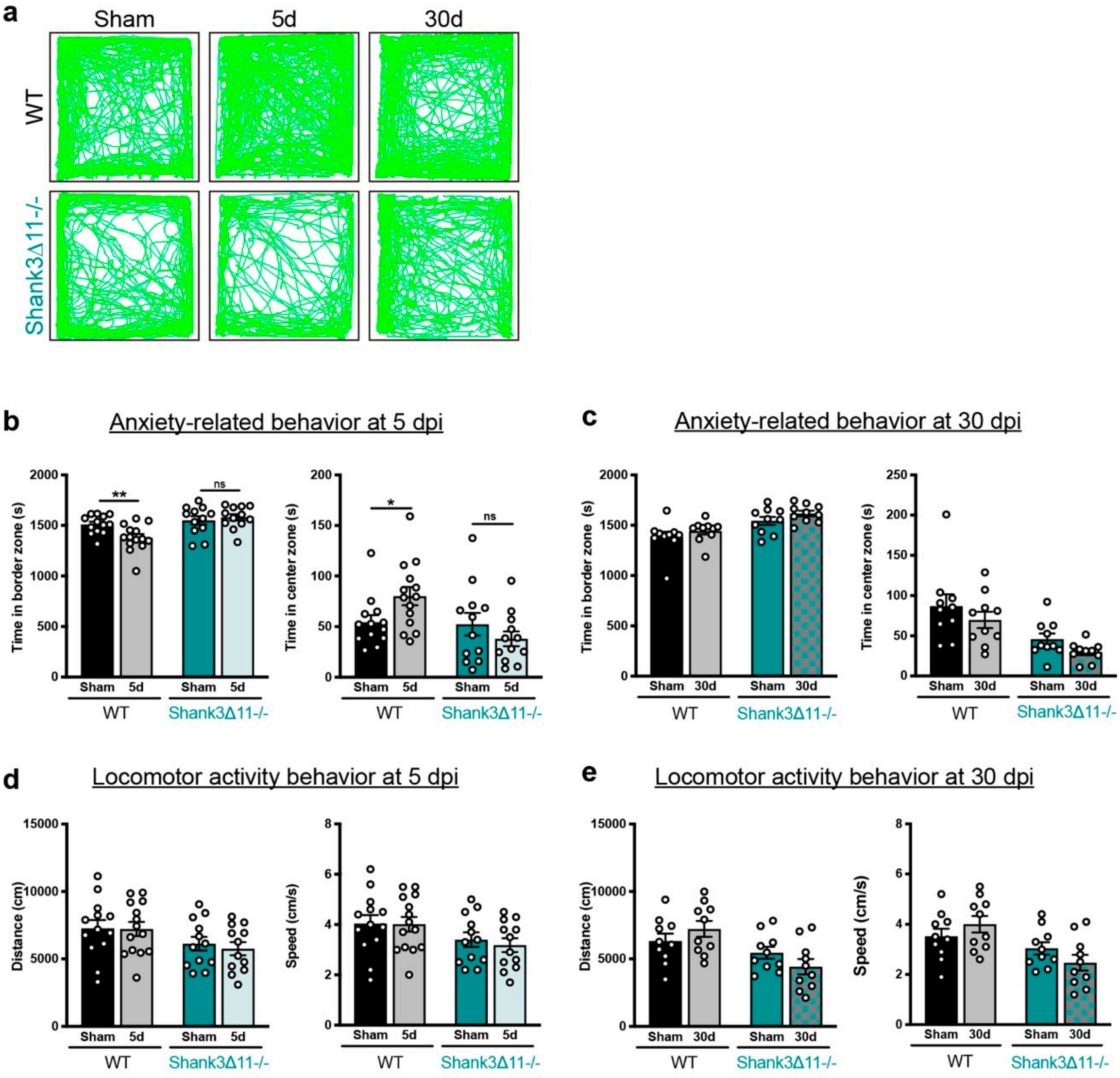

Figure S4. mTBI did not alter the anxious behavior of *Shank3Δ11-/-* mice and the locomotor activity of both wild-type and *Shank3Δ11-/-* mice. (**a**) Representative images of the tracking trajectory of WT (left upper panel) and *Shank3Δ11-/-* mice (left bottom panel) following five and thirty dpi. (**b**) Anxiety-related behavior at five dpi was measured by the time spent on the border or center zone of the arena of WT animals (black bars) and *Shank3Δ11-/-* (turquoise bars) mice. N=12-14. Data are shown as mean ± SEM. Data sets were analyzed using two-way ANOVA (genotype x mTBI) and Bonferroni's post hoc comparison test. [Duration in the border zone at five dpi: genotype F(1, 47) = 12.65, *p* = 0.0009; mTBI F(1, 47) = 2.309, *p* = 0.1353; genotype x mTBI F(1, 47) = 5.805, *p* = 0.0199; duration in the center zone at five dpi: genotype F(1, 47) = 6.435, *p* = 0.0146; mTBI F(1, 47) = 0.4243, *p* = 0.5189; genotype x mTBI F(1, 47) = 5.267, *p* = 0.0262]. (**c**) Anxiety-related behavior at 30 dpi was measured by the time spent on the border or center zone of the arena of WT animals (black bars) and *Shank3Δ11-/-* (turquoise bars) mice. N=10. Data are shown as mean ± SEM. Data sets were analyzed using two-way ANOVA (genotype x mTBI) and Bonferroni's post hoc comparison test. [Duration in the border zone at 30 dpi: genotype F(1, 36) = 17.75, *p* = 0.0002; mTBI F(1, 36) = 2.864, *p* = 0.0992; genotype x mTBI F(1, 36) = 0.03461, *p* = 0.8535; duration in the center zone at 30 dpi: genotype F(1, 36) = 17.44, *p* = 0.0002; mTBI F(1, 36) = 2.918, *p* = 0.0962; genotype x mTBI F(1, 36) = 0.003803, *p* = 0.9512]. (**d**) Assessment of the locomotor activity at five dpi of WT animals (black bars) and *Shank3Δ11-/-* (turquoise bars) mice. N=12-14. Data are shown as mean ± SEM. Data sets were analyzed using two-way ANOVA (genotype x mTBI) and Bonferroni's post hoc comparison test. [Distance at five dpi: genotype F(1, 47) = 5.844, *p* = 0.0196; mTBI F(1, 47) = 0.1477, *p* = 0.7024; genotype x mTBI F(1, 47) = 0.09242, *p* = 0.7625; speed at five dpi: genotype F(1, 47) = 5.932, *p* = 0.0187; mTBI F(1, 47) = 0.1594, *p* = 0.6916; genotype x mTBI F(1, 47)

= 0.1018, $p = 0.7511$]. (**e**) Assessment of the locomotor activity at 30 dpi of WT animals (black bars) and *Shank3Δ11-/-* (turquoise bars) mice. N=12-14. Data are shown as mean ± SEM. Data sets were analyzed using two-way ANOVA (genotype x mTBI) and Bonferroni's post hoc comparison test. [Distance at 30 dpi: genotype $F(1, 36) = 11.57$, $p = 0.0017$; mTBI $F(1, 36) = 0.01526$, $p = 0.9024$; genotype x mTBI $F(1, 36) = 3.188$, $p = 0.0826$; speed at 30 dpi: genotype $F(1, 36) = 11.26$, $p = 0.0019$; mTBI $F(1. 36) = 0.02257$, $p = 0.8814$; genotype x mTBI $F(1, 36) = 3.072$, $p = 0.0882$] (* $p < 0.05$: comparison regarding WT sham animals).

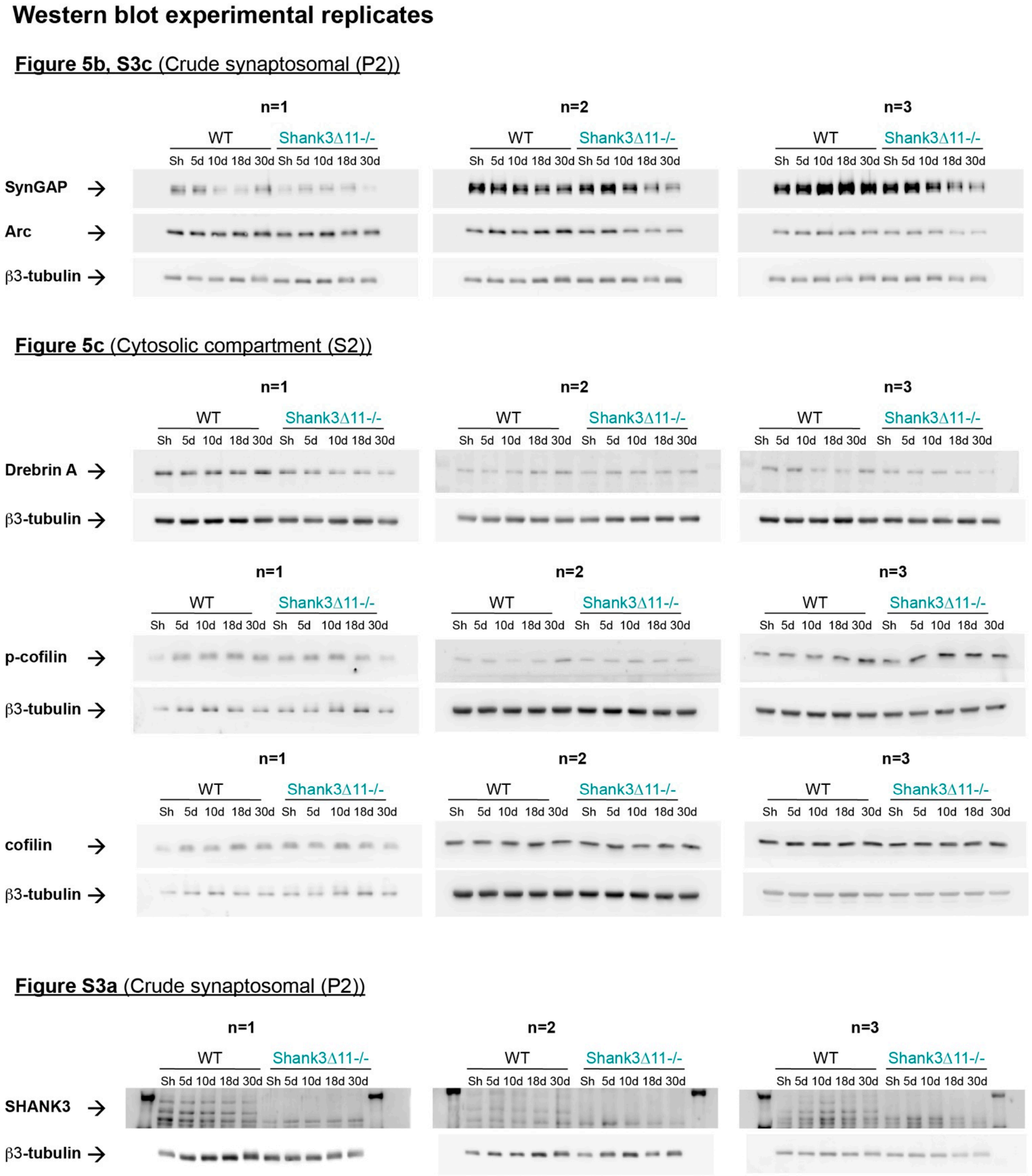

Figure S5. Western blot images for each experimental replicate used in this study. Figures 5b, 5c and 3Sa, c.

Western blot experimental replicates

Figure S3b (Crude synaptosomal (P2))

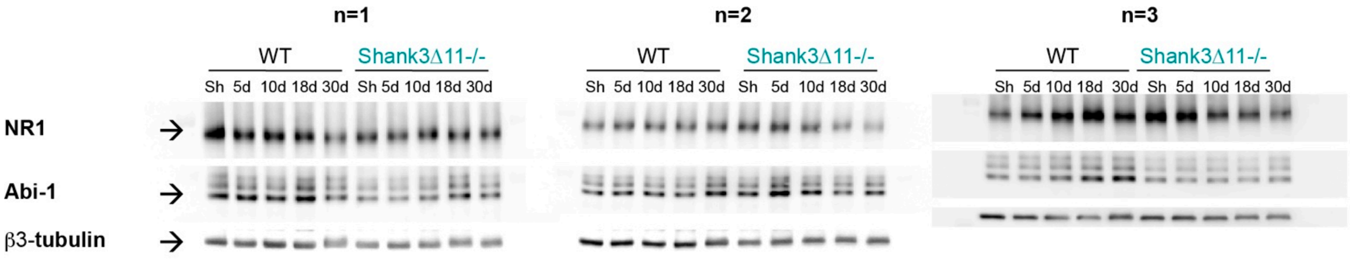

Figure S3d (Crude synaptosomal (P2))

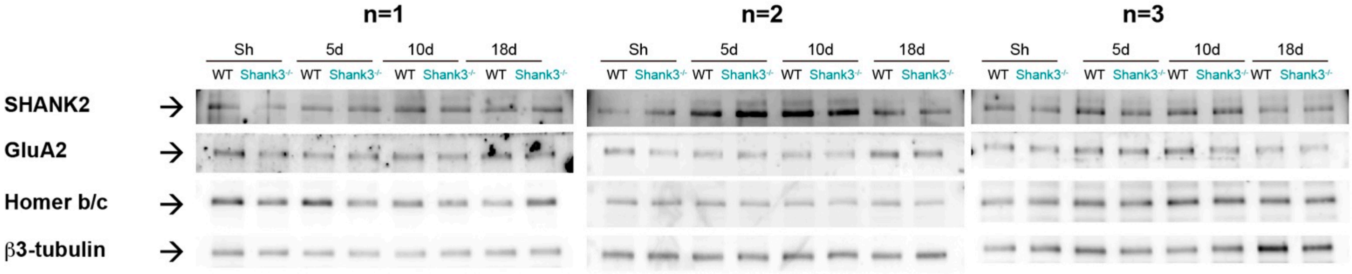

Figure S3e (Crude synaptosomal (P2))

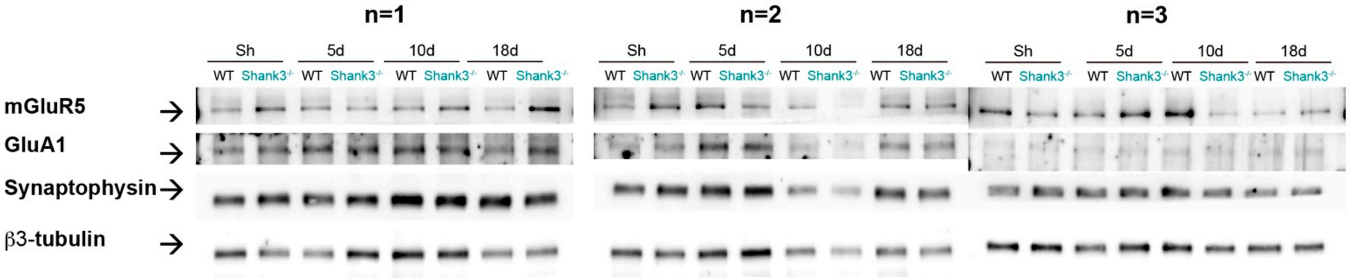

Figure S3f (Cytosolic compartment (S2))

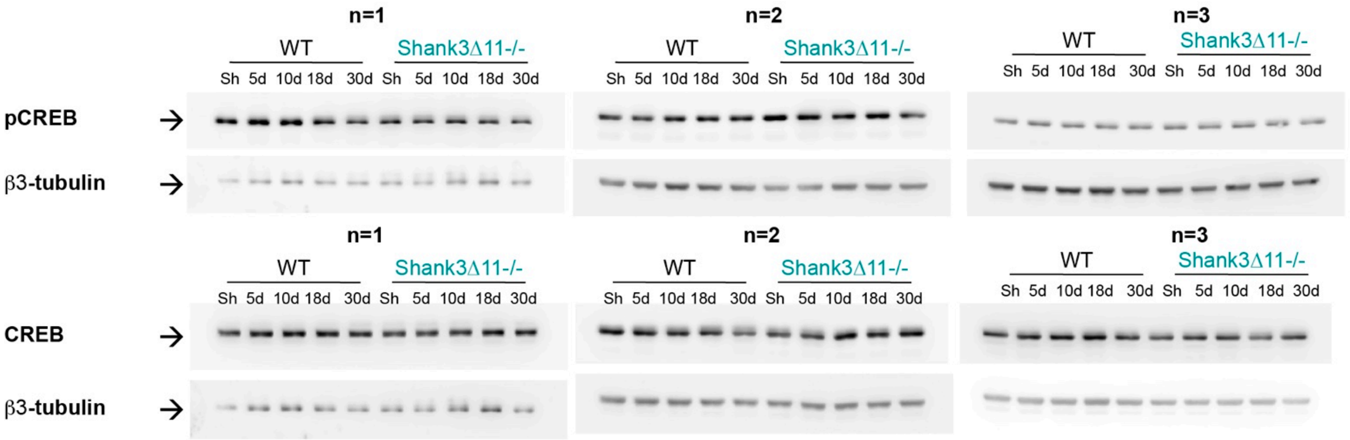

Figure S6. Western blot images for each experimental replicate used in this study. Figures 5Sb,d,e,f.
